# Function-preserving anterolateral thigh flap reconstruction after auricular rhabdomyosarcoma resection in an adolescent: a case report

**DOI:** 10.3389/fped.2026.1780620

**Published:** 2026-03-30

**Authors:** Ryota Koshu, Masao Noda, Yuta Kawahara, Hitomi Niijima, Shino Higai, Ataru Sunaga, Takahiro Fukuhara, Makoto Ito

**Affiliations:** 1 Department of Otolaryngology, Jichi Medical University, Shimotsuke, Japan; 2 Department of Pediatrics, Jichi Medical University, Shimotsuke, Japan; 3 Department of Plastic Surgery, Jichi Medical University, Shimotsuke, Japan

**Keywords:** anterolateral thigh flap, auricular rhabdomyosarcoma, case report, external auditory canal reconstruction, pediatric sarcoma

## Abstract

Auricular rhabdomyosarcoma is an extremely rare malignancy in the pediatric population, and its management presents unique challenges due to both oncologic and reconstructive considerations. Chemotherapy remains the cornerstone of treatment, but surgical intervention is indicated when the tumor demonstrates limited response to induction therapy or when durable local control is required. We report the case of a 16-year-old boy with rhabdomyosarcoma originating in the auricle and extending into the external auditory canal (EAC). He underwent wide local excision of part of the auricle, lateral EAC, and superficial parotid gland after a limited response to induction chemotherapy. Immediate reconstruction of the EAC was performed using a tubularized anterolateral thigh (ALT) flap, with the epithelial surface oriented outward to preserve canal patency and auditory function. Despite the inherent thickness of the ALT flap, this method achieved favorable outcomes, including successful postoperative radiation therapy, preserved hearing, and negative surgical margins. The patient remained disease-free with preserved auditory function and only minimal canal narrowing at the 5-year follow-up visit. This case demonstrates that ALT flap tubularization is a viable reconstructive technique, even for complex pediatric sarcoma cases. It provides a balance between oncologic safety, functional preservation, and long-term durability.

## 1 Introduction

Rhabdomyosarcoma is the most common soft tissue sarcoma in children. It is a high-grade malignant neoplasm characterized by skeletal myoblast-like differentiation (1–3). It may arise in various anatomical locations, and the head and neck region constitutes a substantial proportion of pediatric cases. Tumors originating in the auricle or external auditory canal (EAC) are exceedingly rare and sparsely documented. Standard therapeutic strategies for rhabdomyosarcoma involve multimodal approaches combining cytotoxic chemotherapy and radiotherapy, as well as surgical resection when necessary (2–4). The Intergroup Rhabdomyosarcoma Study Group and subsequent cooperative group trials have emphasized the importance of histologic response and local control in optimizing survival outcomes (4). Patients with residual disease or recurrence after initial therapy have significantly worse prognoses, emphasizing the critical role of complete tumor eradication in long-term survival (5, 6). Surgical interventions for head and neck rhabdomyosarcoma should account for oncologic adequacy and ensure preservation of form and function. Resection involving the auricle and EAC presents a unique reconstructive challenge due to the need for radical excision with negative margins while preserving hearing and accommodating external devices such as eyeglasses or masks (7, 8). Reconstruction of the EAC is crucial for preserving conductive hearing, as maintaining a patent and epithelialized canal prevents complications such as stenosis and chronic otorrhea that can impair hearing outcomes. Local flaps, such as the inferior pedicled square screw flap, have demonstrated effectiveness for small-to-moderate EAC defects by providing straightforward, localized reconstruction with minimal donor site morbidity (9). These flaps can be advantageous for selected cases due to their simplicity and proximity; however, their use may be restricted for more extensive resections due to limited reach and soft tissue volume (10). Microvascular free flaps—especially the anterolateral thigh (ALT) flap—are well-suited for reconstructing complex three-dimensional defects. The ALT flap provides abundant tissue, pliability, and a long vascular pedicle, which are beneficial in pediatric patients requiring wide oncologic resection (11). The ALT flap enables functional reconstruction of the EAC when tubularized. It also maintains hearing and supports both oncologic control and structural integrity.

We report a rare case of auricular rhabdomyosarcoma in a pediatric patient treated with wide local excision and immediate reconstruction of the EAC using a rolled ALT flap. This case illustrates a technically feasible and function-preserving reconstructive strategy for an uncommon tumor location.

## 2 Case description

**Figure 1:**
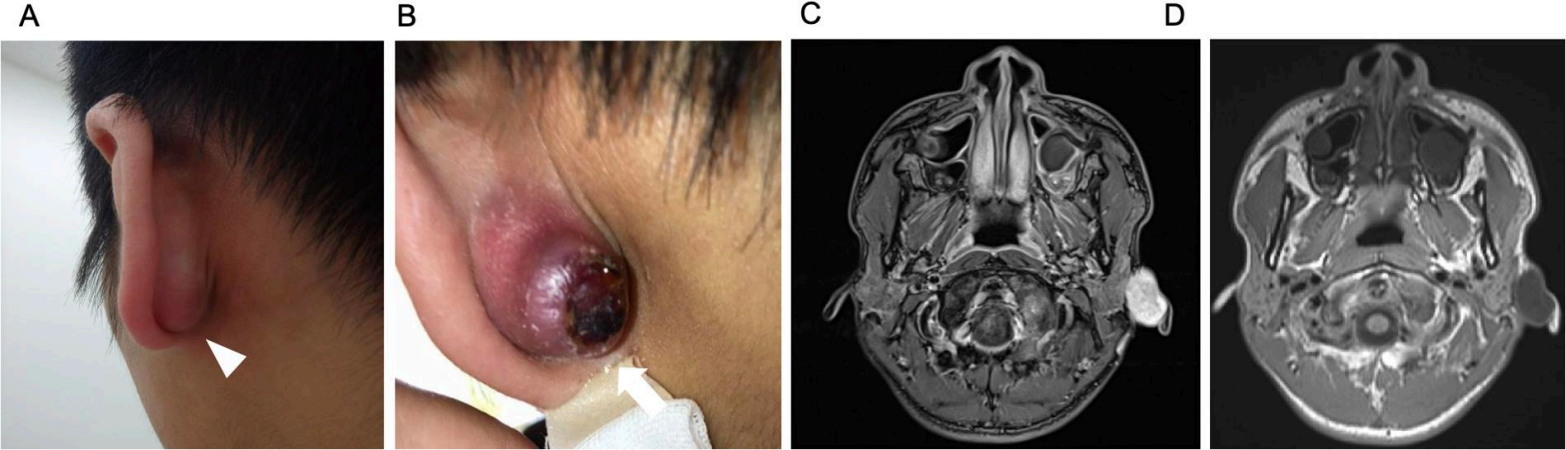
Preoperative clinical and radiologic findings. **(A)** Clinical photograph of the left postauricular region showing a progressively enlarging, painless mass (arrowhead). **(B)** Clinical photograph of the lesion, which had gradually increased in size over approximately 6 months and extended anteriorly to involve the preauricular area. Note overlying skin discoloration and central necrotic area (arrow). **(C)** Shows contrast-enhanced T1-weighted MRI, and **(D)** shows non-contrast T1-weighted MRI.

A 16-year-old boy presented with a painless, progressively enlarging mass in the left postauricular region. The lesion had gradually increased in size over approximately 6 months and had extended anteriorly to involve the preauricular area at the time of initial evaluation (Figures 1A,B). There was no relevant family history of early-onset malignancy or known genetic disorder. Physical examination revealed a firm, non-tender postauricular mass with overlying skin discoloration and a central necrotic area (Figure 1B). Cranial nerve examination, including facial nerve function, was intact. No clinically apparent cervical lymphadenopathy was detected.

Initial MRI revealed a 14.9 × 23.6 × 27.5 mm heterogeneous mass with low signal intensity on T1-weighted images and heterogeneous contrast enhancement. Non-contrast and contrast-enhanced T1-weighted images are shown in Figures 1C,D, respectively. On MRI, the tumor was located in the inferior auricle with limited extension to the inferior wall of the lateral EAC. There was no evidence of invasion into the superior EAC, the tympanic membrane/middle ear, the bony EAC, or the skull base; superficial parotid involvement was suspected.

An incisional biopsy performed under local anesthesia revealed round cells with evidence of skeletal muscle differentiation, consistent with rhabdomyosarcoma. On the biopsy specimen, definitive subclassification (embryonal vs. alveolar) was not possible at the treating institution, and an alveolar component could not be reliably excluded. Based on these findings, the tumor was provisionally managed as alveolar rhabdomyosarcoma.Immunohistochemistry was positive for desmin and myogenin, with additional positivity for *α*-smooth muscle actin (*α*-SMA), muscle-specific actin (MSA), vimentin, CD99, and CAM5.2. MyoD1 and myoglobin were negative. AE1/AE3, EMA, LCA, CD34, CD68, S100, chromogranin A, and synaptophysin were negative. INI1 (BAF47) expression was retained. The Ki−67 labeling index was approximately 80%–90%. Reverse transcription polymerase chain reaction on frozen tissue did not detect PAX3::FKHR or PAX7::FKHR fusion transcripts. Systemic imaging with CT and MRI showed no evidence of distant metastasis, and the tumor was staged as T1aN0M0 (cN0 based on clinical and radiologic assessment).

Accordingly, the patient was classified as intermediate-risk, and chemotherapy was initiated according to the D9803 protocol of the Children's Oncology Group (COG) (12). Induction chemotherapy was initiated using VAC (vincristine, actinomycin D, cyclophosphamide); however, no significant tumor shrinkage was observed. The regimen was subsequently changed to VI (vincristine, irinotecan), but it was also ineffective; thus, surgery was recommended for the patient. Given the limited response to induction therapy and the feasibility of complete resection while preserving function, definitive surgery was pursued to achieve local control.

**Figure 2:**
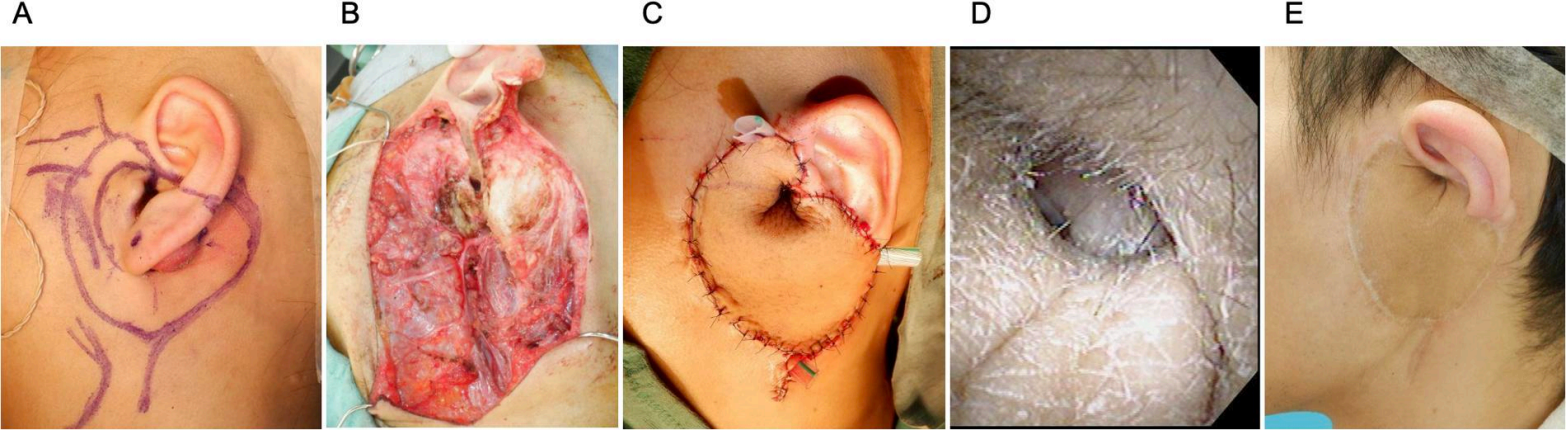
Intraoperative resection and immediate reconstruction with a tubularized anterolateral thigh (ALT) flap. **(A)** Intraoperative photograph after surgical marking, indicating the planned margins around the auricular cartilage and lateral external auditory canal defect. **(B)** View of the composite defect following wide local excision, including partial auricle, lateral canal wall, and superficial parotid tissue; preserved superior canal margin is noted. **(C)** Immediate reconstruction inset showing the ALT skin paddle tubularized with its epithelial surface outward to recreate the external auditory canal, secured to the residual canal edges. **(D)** Endoscopic view of the reconstructed external auditory canal in the early postoperative period, demonstrating a patent, epithelialized lumen with only minimal narrowing at the inferior aspect. **(E)** Clinical photograph at the 5-year follow-up, showing preserved auricular contour and a well-healed scar surrounding the ALT flap inset.

Wide local excision, including the lower posterior auricle and part of the lateral EAC, was performed after completion of two cycles of VAC and two cycles of VI (Figures 2A,B). A macroscopic margin of approximately 1–2 cm was planned where anatomically feasible. In the peri-EAC region, a wider uniform margin was limited by anatomical constraints and the need to preserve hearing-related structures; therefore, an approximately 1 cm margin was used around the EAC. The auricular cartilage was transected nearly horizontally, preserving a 3 mm strip of the superior EAC from the superior aspect of the EAC, corresponding to the 12 to 1 o'clock orientation. The tragus cartilage was also included in the resection. Superficial parotidectomy was performed, and the facial nerve was preserved using intraoperative nerve monitoring. Intraoperative frozen section confirmed negative margins, including those at the EAC, parotid gland, and surrounding skin tissue. Preservation of a narrow superior EAC rim was considered oncologically acceptable because preoperative imaging showed no invasion of the superior EAC or bony canal, and negative EAC margins were confirmed on intraoperative frozen section.

The reconstruction was performed using an ALT flap. The skin paddle was externally tubularized with the epithelial surface oriented outward to reconstruct the EAC (Figure 2C). The ALT flap was inset to the remaining canal edges and sutured in place to ensure continuous epithelialization and structural support. Microvascular anastomosis was performed under an operating microscope in an end-to-end fashion to connect the descending branch of the lateral circumflex femoral artery to the superficial temporal artery and one vena comitans to the superficial temporal vein. Perfusion was confirmed intraoperatively, and no venous congestion was observed.

Although the surgical margins were negative, postoperative radiotherapy (36 Gy in 20 fractions) was delivered as part of the ongoing treatment plan established during the initial intermediate-risk treatment course. Final central pathology results became available only after radiotherapy had already started, at which point the diagnosis was definitively established as embryonal rhabdomyosarcoma and the patient was reclassified as low-risk subgroup B. Subsequent systemic therapy was then continued within a COG risk-adapted framework, with reference to prior low-risk subgroup B protocols (13). During subsequent vincristine-containing chemotherapy, the patient developed peripheral neuropathy affecting activities of daily living, leading to temporary discontinuation of vincristine. In order to preserve treatment intensity while decreasing the frequency of vincristine administration, the planned three cycles of VAC were replaced with two cycles of VDC/IE (vincristine, doxorubicin, cyclophosphamide/ifosfamide, etoposide). Neuropathy improved during VDC/IE, after which four additional courses of VAC were administered to complete treatment.

He demonstrated no evidence of disease recurrence and had an intact EAC without significant complications, with preserved hearing and near-normal thresholds at the 5-year follow-up (Figures 2D,E). The reconstructed canal remained patent with only minimal narrowing, and no clinically significant otorrhea or infections were observed during follow-up. The patient and guardian expressed a strong preference for preserving auricular contour and hearing function and agreed with the proposed function-preserving approach. Treatment-related neuropathy improved after regimen modification, and no flap compromise occurred (Table 1).

**Table 1:** Timeline of the episode of care.

Time from presentation	Key events
Month 0	Onset of painless postauricular mass
Month 6 (approximately)	Initial evaluation; MRI performed; biopsy consistent with rhabdomyosarcoma
Induction period	VAC initiated → limited response; VI administered → limited response
Definitive local therapy	Wide local excision including partial auricle, lateral EAC, superficial parotidectomy; negative margins confirmed on frozen section; immediate tubularized ALT flap reconstruction
Adjuvant therapy	Chemotherapy individualized because of limited response and toxicity; radiotherapy 36 Gy in 20 fractions
Long-term follow-up	Disease-free; preserved hearing; minimal canal narrowing at 5-year follow-up

## 3 Discussion

This case involved a pediatric patient with auricular rhabdomyosarcoma that extended into the EAC. Embryonal rhabdomyosarcoma is generally associated with a favorable prognosis, but rapid tumor growth and poor response to chemotherapy may necessitate surgical resection. Comprehensive planning is essential to achieve oncologic control while preserving function and appearance, especially for cases involving anatomically complex regions like the EAC.

Regarding histologic subtype and risk stratification, the diagnosis of embryonal rhabdomyosarcoma was definitively established by central pathology review. Based on the disease extent (T1aN0M0), a favorable non-parameningeal head and neck site, and complete resection with negative margins (IRS Clinical Group I), this presentation corresponds to a low-risk category in the COG risk stratification framework for embryonal rhabdomyosarcoma (2, 13). However, the final central pathology findings were obtained only after radiotherapy had already begun; thus, postoperative radiotherapy was administered as part of the previously initiated intermediate-risk treatment course, whereas subsequent chemotherapy was continued according to a low-risk treatment approach consistent with the D9602 study. The overall treatment course was further individualized because of the limited response to induction chemotherapy and treatment-related toxicity, with the overarching goal of durable local control (5, 6).

The head and neck region poses reconstructive challenges due to its complex anatomy and functional significance. Surgical excision involving the EAC must be accompanied by reconstruction to ensure canal patency, minimize the risk of stenosis and otorrhea, and preserve conductive hearing (14–16). Timely healing is also essential for administering postoperative radiotherapy, which is often required in rhabdomyosarcoma management (17, 18).

Regarding the extent of resection and margin strategy, a uniform centimeter-based margin is not always feasible in the auricle–EAC–parotid region because a superficial linear distance does not reliably represent deep anatomic margins. Therefore, we planned a macroscopic 1–2 cm margin where feasible and prioritized *en bloc* resection along anatomic boundaries; oncologic adequacy was confirmed with intraoperative frozen-section assessment of the skin, EAC, and parotid interfaces. This approach achieved negative margins while preserving critical structures for hearing and reconstruction.

Justification for preserving a narrow superior EAC rim involved maintaining an approximately 3 mm segment of the superior EAC to provide a stable and continuous structural ring. This preserved rim contributed to maintaining the native contour of the ear canal and created a reliable framework for secure inset of the tubularized flap during reconstruction. This was considered oncologically acceptable because preoperative imaging showed no invasion of the superior EAC or bony canal, and negative EAC margins were confirmed intraoperatively.

In considering alternative surgical strategies, lateral temporal bone resection (LTBR) is frequently discussed in cases of malignancies involving the EAC, particularly when there is radiologic or clinical evidence of tumor extension into the bony canal, middle ear, or deeper temporal bone structures. A treatment strategy based on bony invasion has been clearly described for EAC malignancies, in which sleeve resection is used for localized disease without bony invasion, whereas LTBR is selected when bony invasion is present (19). In contrast, our case showed no radiologic evidence of bony EAC, middle ear, or skull base extension, permitting a function-preserving lateral EAC resection while maintaining negative margins. Thus, a less extensive approach was justified to balance oncologic safety with long-term auditory function.

The extent of resection, which included the auricular cartilage, lateral EAC, and superficial parotid gland, precluded the use of local flaps. Various flap types have been explored for reconstructing the EAC, including local flaps and skin grafts (20). Local flaps offer aesthetic advantages due to color matching, but are typically insufficient for extensive defects. A microvascular free flap was selected for its flexibility and capacity to provide adequate soft tissue volume.

The ALT flap was deemed the most suitable option for this young patient due to its abundant tissue, stable vascularity, and low donor-site morbidity. The flap was tubularized intraoperatively to recreate the EAC structure, despite its inherent thickness, which is generally considered suboptimal for canal reconstruction. This technique proved effective in maintaining canal patency and facilitating timely postoperative radiation. Similar rolled flap techniques have been reported for EAC carcinoma (21); however, their successful application in this sarcoma case underscores the broader utility of such approaches. Preserving a portion of the auricle contributed to enhanced functional and cosmetic outcomes and highlights the adaptability of this method across different histologic tumor types.

Alternative options, such as SCIP flaps, have been shown to be useful for thin flap reconstruction. However, the ALT flap was favored for its adequate volume, radiation resilience, and widespread familiarity across institutions (18, 22). Its large-caliber vascular supply supports consistent perfusion and mitigates the risk of necrosis or fibrosis due to radiation exposure.

The patient maintained excellent auditory outcomes, with only minor canal narrowing observed at long-term follow-up. Skin color mismatch remained unresolved, which is typical of free flap reconstruction. Secondary corrective procedures were not pursued due to limited benefits despite the concerns of the patient. This approach facilitated complete tumor resection while preserving hearing and auricular structure. The case supports the strategic use of tubularized ALT flaps in pediatric patients requiring extensive resection for auricular rhabdomyosarcoma to balance oncologic, functional, and cosmetic considerations.

This case highlights a rare instance of auricular rhabdomyosarcoma in a pediatric patient that required wide local excision, including the EAC. Tubularization of the ALT flap allowed for successful reconstruction of the canal while preserving auditory function and enabling timely postoperative radiotherapy despite its typical thickness. Long-term follow-up demonstrated oncologic control, preserved hearing, and stable canal morphology. Minor aesthetic concerns remained, but the overall outcome highlights the ALT flap as a reliable reconstructive option for complex pediatric head and neck sarcoma cases requiring both radical resection and functional restoration.

## 4 Conclusions

This case demonstrates that tubularized ALT flap reconstruction after wide excision of pediatric auricular rhabdomyosarcoma achieves durable oncologic control and preserves auditory function. Despite its thickness, orienting the epithelial surface outward maintained EAC patency, permitted timely radiotherapy, and resulted in only minimal stenosis at 5 years. The ALT flap's reliable vascularity, ample volume, and low donor-site morbidity make it a valuable option for complex head and neck defects in children. Tubularized ALT flaps should be considered when radical resection and functional restoration are both priorities in rare pediatric EAC tumors.

## Data Availability

The datasets presented in this article are not readily available because the dataset contains personal health information and is therefore not publicly available. Data may be provided by the corresponding author upon reasonable request and with appropriate ethical considerations. Requests to access the datasets should be directed to Masao Noda, mnoda@jichi.ac.jp.
